# Sensitive Electrochemical Determination of Vanillin Using a Bimetallic Hydroxide and Reduced Graphene Oxide Nanocomposite

**DOI:** 10.3390/s25061694

**Published:** 2025-03-09

**Authors:** Shamim Ahmed Hira, Jonathan Quintal, Aicheng Chen

**Affiliations:** Electrochemical Technology Centre, Department of Chemistry, University of Guelph, 50 Stone Road East, Guelph, ON N1G 2W1, Canada; shira02@uoguelph.ca (S.A.H.); quintalj@uoguelph.ca (J.Q.)

**Keywords:** vanillin, food safety, square wave voltammetry, limit of detection

## Abstract

Vanillin (VAN) is an organic compound which not only functions as a flavoring and fragrance enhancer in some foods but also has antioxidant, anti-inflammatory, anti-cancer, and anti-depressant effects. However, the excessive use of VAN can be associated with negative side effects on human health. As a result, it is crucial to find a reliable method for the rapid determination of VAN to enhance food safety. Herein, we developed a sensor using Ni and Co bimetallic hydroxide and reduced graphene oxide nanostructure (NiCo(OH)_2_.rGO). Our prepared material was characterized using various physico-chemical techniques. The electrocatalytic efficiency of the NiCo(OH)_2_.rGO-modified glassy carbon electrode was investigated using cyclic and square wave voltammetry. The developed sensor showed a limit of detection of 6.1 nM and a linear range of 5–140 nM. The synergistic effect of NiCo(OH)_2_ and rGO improved the active sites and enhanced its catalytic efficiency. The practical applicability of the prepared sensor was investigated for the determination of VAN in food samples such as biscuits and chocolates, showing promise in practical applications.

## 1. Introduction

Vanillin (VAN), also known as 4-hydroxy-3-methoxybenzaldehyde, is an important chemical component of natural vanilla. Due to its desirable flavour, it is frequently used as a flavouring agent in foods, sweets, beverages, and different pharmaceutical products. It is also utilized as an antioxidant and antimicrobial agent against the growth of bacteria, yeast, and mould [1,2,3]. However, the excessive intake of VAN can cause allergic reactions, headaches, nausea, vomiting, and damage to the liver and kidney [4,5]. Vanillin has been shown to induce a persistent appetite, encouraging its overconsumption [6,7]. The maximum dosage of VAN is 7 mg/100 g for adults, while in infant food, VAN is forbidden [8]. Therefore, from the viewpoint of food safety and addiction prevention, particularly in growing children, it is crucial to develop a selective, sensitive, fast, and simple method for the detection of VAN in foods and beverages.

Electrochemical sensors play an essential role for maintaining food safety through the quantification of potentially harmful substances [9]. Until now, various useful but time-consuming and costly methods have been reported for the detection of VAN, including colorimetry [10], high-performance liquid chromatography [11], fluorescence [12,13], spectrophotometry [14], capillary electrophoresis [15], gas chromatography [16], gas chromatography–mass spectrometry (GC-MS) [17], nuclear magnetic resonance spectrometry [18], and surface-enhanced infrared absorption spectroscopy [19]. Compared to these methods, electrochemical techniques have the advantages of being simultaneously inexpensive, fast, selective, and sensitive [20]. However, reducing the overpotential and maximizing the selectivity and sensitivity requires careful design of the sensor through modifications to the underlying material [21,22,23,24,25,26].

Recently, binary metal-based materials have received a lot of attention as electrocatalysts owing to their superior intrinsic catalytic activity [27,28,29,30]. Among the various metal-based materials which have been introduced for sensor applications, Co- and Ni-containing materials have attracted significant interest due to their excellent catalytic activity, low cost, and strong chemical stability [31]. However, further modification is necessary to improve their overall performance. Until now, many strategies have been followed to increase the electrocatalytic activity of metal hydroxide materials. These include (but are not limited to) the modulation of functional sites through morphology control, the introduction of heteroatoms, defects, and the incorporation of carbon materials to improve electrical conductivity [32,33,34]. Although there have been a few reports on the synthesis of Co-Ni bimetallic materials and rGO composites for energy applications [35,36], no reports were found where bimetallic Co-Ni materials and reduced graphene oxide (rGO) nanostructures were used as a sensor for the electrochemical determination of VAN.

Herein, we report a nanocomposite based on a Ni and Co bimetallic hydroxide, NiCo(OH)_2_, anchored on rGO, and explore its catalytic activity as a selective and sensitive material for the electrochemical determination of VAN. The synthesized material was characterized using various physico-chemical techniques to understand its catalytic activity. Due to the synergistic effect of NiCo(OH)_2_ and rGO, the sensor demonstrated in the present study exhibited high performance for the electrochemical determination of VAN with high selectivity, high sensitivity, and a very low detection limit. The practical utility of our developed electrochemical sensor was further evaluated for the detection of VAN in biscuit and chocolate samples.

## 2. Materials and Methods

### 2.1. Chemicals

VAN (98%), cobalt nitrate hexahydrate (Co(NO_3_)_2_.6H_2_O, ≥97%), nickel nitrate hexahydrate (Ni(NO_3_)_2_.6H_2_O, ≥97%), 2-methylimidazole (2-MeIm, ≥99%), disodium phosphate (Na_2_HPO_4_), monosodium phosphate (NaH_2_PO_4_), and methanol (MeOH, 99%) were obtained from Sigma-Aldrich Co. Ltd. (Oakville, ON, Canada). Phosphate-buffered saline (PBS, pH = 7.2) was prepared by mixing a standard stock solution of 0.2 M of Na_2_HPO_4_ and NaH_2_PO_4_. All reagents were utilized as received without any further purification.

### 2.2. Synthesis of ZIF-67

ZIF-67 was synthesized according to the previously reported method with slight modification [37]. Briefly, 1 mmol of Co(NO_3_)_2_.6H_2_O was put in a round-bottom flask that contained 25 mL MeOH. Separately, 5 mmol of 2-MeIm was added to a conical flask containing 25 mL MeOH. The two solutions were then mixed with stirring for 2 h at 27 °C. The precipitate was collected by centrifugation and washed twice with EtOH and H_2_O. The obtained product was denoted as ZIF-67.

### 2.3. Synthesis of NiCoZIF-67

The NiCoZIF-67 was synthesized using a hydrothermal method in accordance with a previously reported procedure but with minor modifications [37]. First, 1 mmol Ni(NO_3_)_2_.6H_2_O and 1 mmol of Co(NO_3_)_2_.6H_2_O were mixed in a round-bottom flask that contained 25 mL MeOH. Separately, 5 mmol of 2-MeIm was prepared in a conical flask containing 25 mL MeOH and mixed. The two solutions were then mixed with stirring for 2 h at 27 °C. The precipitate was collected by centrifugation and washed two times with EtOH and H_2_O. The material prepared in that way was denoted as NiCoZIF-67, with a yield of 73%.

### 2.4. Synthesis of GO

The GO was synthesized using a modified Hummer’s method [38]. Firstly, 1.0 g graphite, 90.0 mL sulfuric acid (H_2_SO_4_), and 10.0 mL phosphoric acid (H_3_PO_4_) were added to a reactor vessel. Next, the mixture was stirred vigorously for 4 h at 50 °C. Then, 4.5 g of KMnO_4_ was added and the whole mixture was stirred continuously for another 15 h. Finally, 100 mL of ice water was added to the mixture, followed by the addition of 5.0 mL of 30% H_2_O_2_. The GO was separated and washed with H_2_O and EtOH. The as-synthesized GO was dried in an oven at 50 °C.

### 2.5. Synthesis of NiCo(OH)_2_.rGO

First, 1 mmol Ni(NO_3_)_2_.6H_2_O and 1 mmol of Co(NO_3_)_2_.6H_2_O were added to a round-bottom flask that contained 25 mL MeOH and dispersed properly. Next, 5 mg GO was added to 5 mL H_2_O and sonicated to produce a uniform dispersion, and then the mixture was stirred for 30 min. Then, 5 mmol of 2-MeIm was prepared in a conical flask containing 25 mL MeOH and mixed. Both solutions were mixed together with stirring for 2 h at 27 °C. The precipitate was collected by centrifugation and washed twice with EtOH and H_2_O. The prepared sample was denoted as NiCo(OH)_2_.rGO with a yield of 68%.

### 2.6. Characterization Techniques

The physical characterization of the synthesized materials was conducted using a variety of techniques, including field emission scanning electron microscopy (FE-SEM), energy dispersive X-ray spectroscopy (EDX), and X-ray photoelectron microscopy (XPS). The SEM images were obtained using a FEI Quanta FEG 250. The EDX data were collected using an Oxford XMax20 spectrometer with Aztec 2.1 software. The XPS data were collected using a Scienta Omicorn spectrometer, with excitation from an Al Kα X-ray source with a 15 mA emission current. The XPS samples were run on an insulating tape and charge corrected using the C1s spectrum. The XPS analysis was conducted using CasaXPS Version 2.3.24PR1.0.

### 2.7. Electrochemical Measurement

All the electrochemical measurements were carried out utilizing a CHI700E CH electrochemical workstation. A traditional three-electrode system was applied for the electrochemical determination, where a modified GCE was used as the working electrode, Pt as the counter electrode, and Ag/AgCl as the reference electrode. The PBS buffer was deoxygenated before each electrochemical experiment by purging it with high-purity Ar gas.

### 2.8. Preparation of the Electrode

First, 0.5 mg of NiCo(OH)_2_.rGO was added to a vial containing 160 μL of a solution which contained a 1:7:7 ratio of Nafion, H_2_O (MilliQ, 18.2 ΜΩ), and EtOH. The mixture was then sonicated to produce a homogenous dispersion. Prior to modification, the GCE was polished using a slurry of aluminum powder and water and then washed with a 1:1:1 ratio of EtOH, acetone, and H_2_O, before being dried at 22 °C. Then, 3 µL of the NiCo(OH)_2_.rGO ink was drop-cast onto the surface of the GCE and allowed to dry at 22 °C for 3 h. The as-prepared electrode was utilized for the electrochemical determination of VAN. A similar procedure was followed to prepare the other modified electrodes.

## 3. Results and Discussion

### 3.1. Physical Characterization

**Figure 1:**
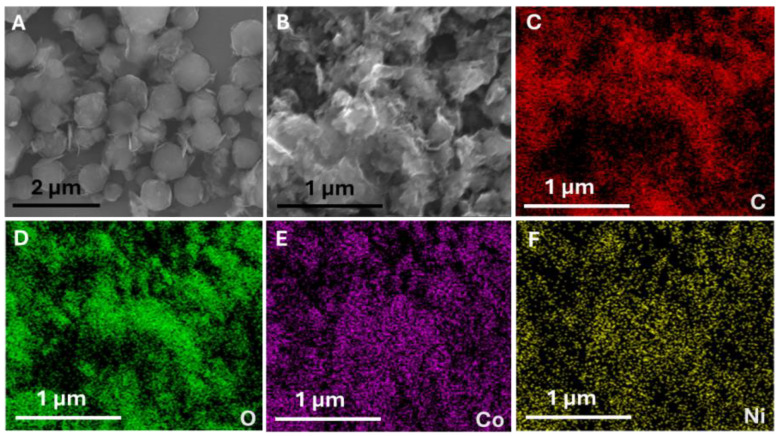
SEM image of NiCo-ZIF-67 (**A**), SEM image (**B**) and elemental mapping for carbon (**C**), oxygen (**D**), cobalt (**E**), and nickel (**F**) of NiCo(OH)_2_.rGO.

FE-SEM was utilized to investigate the morphology of synthesized materials. NiCoZIF-67 exhibited a dodecahedral morphology with uniform dimensions (Figure 1A). However, the morphology was observed to change in the formation of NiCo(OH)_2_.rGO, which showed a chestnut-like structure (Figure 1B). The difference in structure upon the addition of GO to the synthesis was evident from the SEM images, and the characteristic polygonal shapes associated with ZIF-67 were absent. This may suggest a significant change in the chemical composition of the material itself. Elemental mapping (Figure 1C–F) showed the existence of C, O, Co, and Ni.

**Figure 2:**
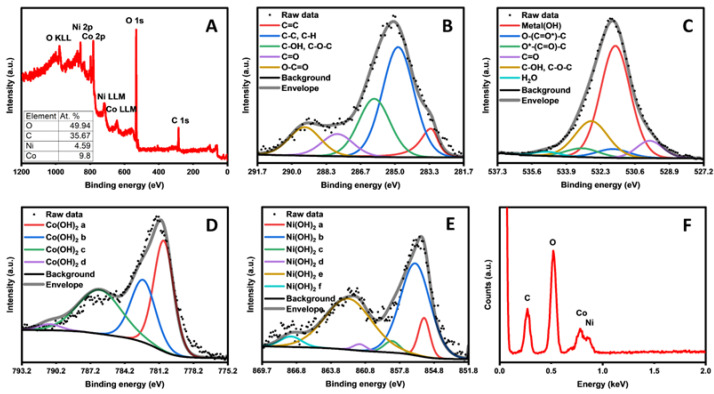
XPS survey spectrum (**A**), high-resolution XPS spectrum of carbon (**B**), oxygen (**C**), cobalt (**D**), and nickel (**E**), EDS spectra (**F**) of NiCo(OH)_2_.rGO.

XPS was also employed to investigate the chemical states present in the sensor material. The XPS survey spectrum, displayed in (Figure 2A), showed photoemission peaks from C 1s, O 1s, Co 2p and Ni 2p at approx. 284.5, 532.5, 780.9, and 856.5 eV binding energies, respectively. Additionally, two auger electron emission peaks were observed for the Co and Ni LLM auger process at 715 and 641 eV, respectively. From the photoemission peaks, the atomic percentages of each element were determined to be 49.94% O, 35.67% C, 4.59% Ni, and 9.80% Co, as shown in the table (inset of Figure 2A). The absence of a peak associated with nitrogen was indicative of the methylimidazole not being incorporated into the structure. Further investigation of the binding environments for these elements was conducted using high-resolution XPS of the primary emission peaks for the listed elements. The high-resolution spectra for carbon, shown in Figure 2B, contained typical graphene functional groups and were dominated by a high amount of sp^3^ carbon content. The precursor material and primary carbon source for this sensor was graphene oxide, so the lack of a large carbon–oxygen contribution to this peak was indicative of the reduction of GO to rGO, likely caused by 2-methylimidizole acting as a reducing agent, which could explain the lack of ZIF formation. The high-resolution oxygen peak, shown in Figure 2C, contained typical graphene functional groups in low quantities, consistent with the carbon 1s spectra. This peak was dominated by a large metal hydroxide peak, indicating that the nickel and cobalt present in the sample were likely in the form of a metal hydroxide. These data are in line with the conclusion that the addition of GO in the synthesis has an inhibitory effect on the formation of the ZIF structure, preferring to form rGO-supported metal hydroxides instead. The high-resolution Co 2p XPS also showed marked differences with the ZIF-67 reported in the literature. The high-resolution cobalt spectrum, fitted using a standard set of peak models [39], best matched the expected line shape of cobalt (II) hydroxide. The high-resolution nickel spectrum was fitted in a similar manner and showed a best match with nickel (II) hydroxide [39]. These spectra are shown in Figure 2D,E, respectively. These hydroxides were formed in the alkaline environment during the single-pot synthesis. The XPS results agreed with the EDS spectra (Figure 2F).

### 3.2. Electrochemical Studies

**Figure 3:**
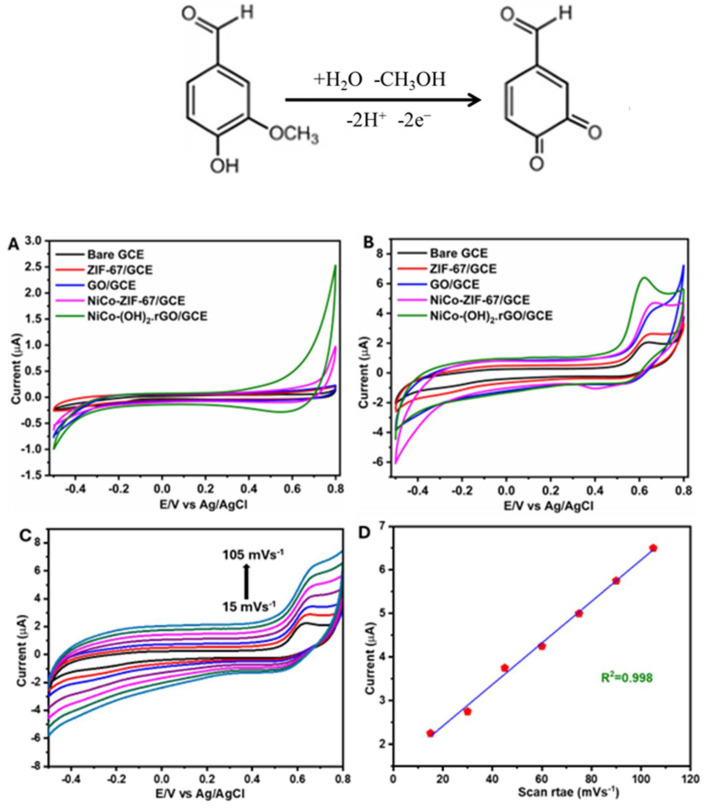
Cyclic voltammograms of all electrodes in PBS (pH = 7.2) in the absence (**A**), presence of 140 nM vanillin (**B**), cyclic voltammogram of NiCo(OH)_2_.rGO/GCE in PBS (pH = 7.2) with various scan rate (mVs−1) (**C**), and correlation between peak current and scan rate (**D**).

The electrochemical response of VAN on different modified electrodes was investigated using cyclic voltammetry (CV). Figure 3A shows the cyclic voltammograms (CVs) of all the electrodes recorded in PBS (pH = 7.2) in the absence of VAN. No notable peaks were observed in any of the electrodes but the current signal increased significantly on NiCo(OH)_2_.rGO/GCE compared to all other electrodes. Figure 3B displays the cyclic voltammograms of all electrodes in PBS (pH = 7.2) in the presence of 140 nM VAN. The experiments were conducted at a scan rate of 50 mVs^−1^ with a fixed potential window of −0.5 to 0.8 V vs. Ag/AgCl. The anodic response was associated with the oxidation of the VAN, and the cathodic peak in the reverse scan correlated with the reduction of the catechol unit. The associated oxidation scheme can be illustrated as follows [40]:

The anodic current response for ZIF-67/GCE (red curve) was higher than that of a bare GCE (black curve). A further increase in current was observed for GO/GCE (blue curve) and NiCoZIF-67/GCE (pink curve). The highest current was achieved at NiCo(OH)_2_.rGO/GCE (green curve), which also occurred at a noticeably lower peak potential. This indicates that there is likely a lower activation energy barrier towards the oxidation of VAN when compared with the other electrodes. Furthermore, the synergistic effect of NiCo(OH)_2_ and rGO increases the active surface area and results in faster electron transfer and superior electrocatalytic activity due to interactions between VAN and rGO. A scan rate study was performed to determine the kinetic behaviour of NiCo(OH)_2_.rGO/GCE towards the electrochemical determination of VAN. Figure 3C shows the electrochemical oxidation of VAN at NiCo(OH)_2_.rGO/GCE in PBS (pH 7.2) at various scan rates (15–105 mVs^−1^). The anodic current increased with the increasing scan rate. The relationship between the anodic current and the scan rate is shown in Figure 3D, which gives a linear response with R^2^ value of 0.998. This relationship indicates that the oxidation of VAN is an adsorption-controlled process [41].

**Figure 4:**
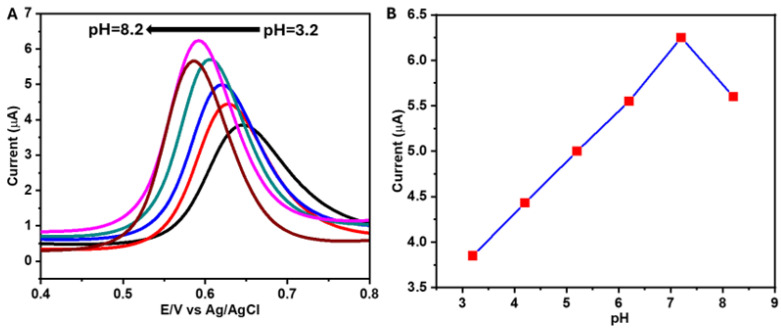
SWVs of NiCo(OH)_2_.rGO/GCE for the electrochemical detection of VAN (140 nM) in PBS with various pH starting from pH = 3.2 to pH = 8.2 at an amplitude of 50 mV and frequency of 25 Hz (**A**), plot of pH vs. peak current (**B**).

The pH of the electrolyte solution has a significant effect on the electrochemical response of VAN. Figure 4A shows the square wave voltammogram (SWV) curves of VAN using NiCo(OH)_2_.rGO/GCE with various pH values, where the highest current response was obtained at pH = 7.2. This was largely due to the pKa of vanillin being approximately 7.9. The deprotonation of the alcohol proton was likely responsible for the decreased reaction kinetics, which can be seen in a reduction in the current associated with the formation of the oxidation product. As the pH increased from 3.2 to 8.2, the peak potential shifted lower, indicating the participation of protons in the oxidation process, while the decreasing current represented reduced involvement of the protons [42,43]. pH 7.2 was observed to be the optimal electrolyte pH for the electrochemical detection of VAN (Figure 4B).

**Figure 5:**
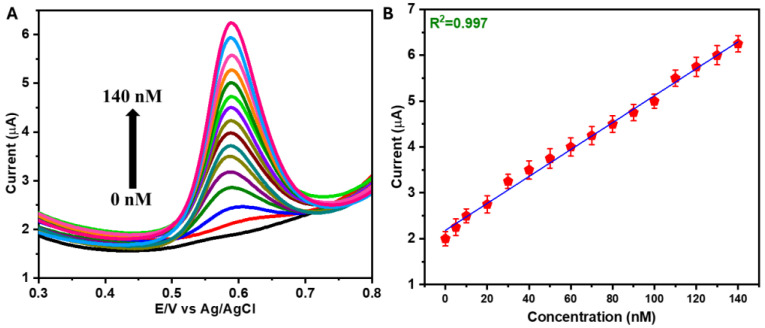
SWVs of NiCo(OH)_2_.rGO/GCE in PBS (pH = 7.2) in the presence of different concentrations of VAN (down to up: 0 nM to 140 nM) at an amplitude of 50 mV and frequency 25 Hz (**A**) and the linear plot of current vs. concentration (**B**).

Figure 5A shows the SWV of NiCo(OH)_2_.rGO/GCE for the detection of VAN, with various concentrations from 0 nM to 140 nM in PBS (pH 7.2). The oxidative current was observed to be proportional to the concentration of VAN, and our prepared sensor exhibited good sensitivity, even at low concentrations. Figure 5B displays the linear relationship between the current response and VAN concentration. A very low limit of detection (LOD = 6.1 nM) was obtained for the determination of VAN. The LOD is calculated using the equation LOD = 3S/b, where S represents the standard deviation of the current response for five measurements, and b is the slope of the calibration curve. The LOD value of the proposed sensor was lower than those of the previously reported VAN sensors (Table 1).

### 3.3. Selectivity, Reproducibility, and Stability

**Figure 6:**
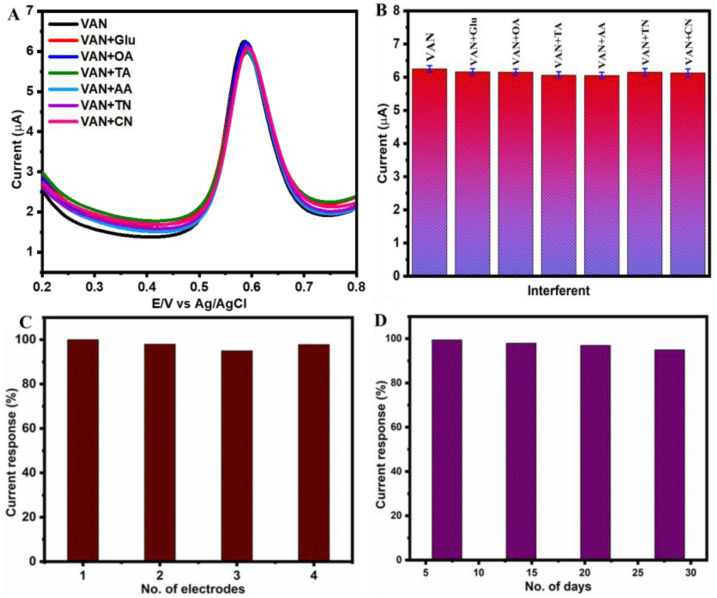
SWV response of NiCo(OH)_2_.rGO/GCE in PBS (pH = 7.2) at an amplitude of 50 mV and frequency of 25 Hz for the electrochemical detection of VAN (140 nM) with 25-fold concentration (3.5 µM) of various organic interferences such as glucose (Glu), oxalic acid (OA), tartaric acid (TA), ascorbic acid (AA), tartrazine (TN), and curcumin (CN) (**A**), anti-interference property of NiCo(OH)_2_.rGO/GCE using bar diagram (**B**), reproducibility of NiCo(OH)_2_.rGO/GCE in the presence VAN (140 nM) with four GCE electrodes (**C**), stability of NiCo(OH)_2_.rGO/GCE as its continuous usage for 28 days containing VAN (140 nM) in PBS (pH = 7.2) (**D**).

To ensure the practical application of the designed sensor (NiCo(OH)_2_.rGO/GCE), selectivity, reproducibility, and stability were considered as essential parameters. The selectivity of NiCo(OH)_2_.rGO/GCE was investigated for the electrochemical detection of VAN in the presence of a high concentrations (25 times the VAN concentration) of organics typically found in food products such as glucose (Glu), oxalic acid (OA), ascorbic acid (AA), tartaric acid (TA), curcumin (CN), and tartrazine (TN). Figure 6A,B show no significant changes in the current for the detection of VAN in the presence of high concentrations of possible interfering agents, which indicates that our sensor is highly selective to VAN compared to some other possible interferents.

To test for reproducibility, four electrodes were prepared in identical conditions using NiCo(OH)_2_.rGO/GCE, and their electrochemical activities towards the detection of 140 nM VAN were measured. The oxidative peak current obtained from four independent electrodes exhibited excellent reproducible results with relative standard deviations of 4.2%, confirming the reproducibility of our results towards the electrochemical detection of VAN, as shown in Figure 6C. Figure 6D shows the storage stability of NiCo(OH)_2_.rGO/GCE over 28 days; the retention percentage was obtained as 99.5%. The RSD value was calculated as 2.35%. The acceptable RSD value proved the suitability of our designed sensor for the electrochemical determination of VAN.

### 3.4. Real Sample Analysis

**Figure 7:**
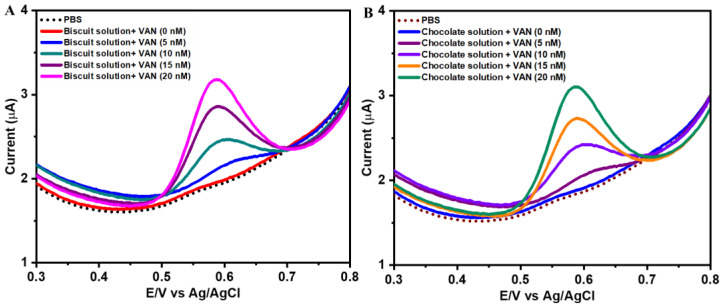
SWV response of NiCo(OH)_2_.rGO/GCE in PBS (pH = 7.2) at an amplitude of 50 mV and frequency 25 Hz for the 0, 5, 10, 15 and 20 nM vanillin in biscuit samples (**A**) and chocolate samples (**B**).

The practical applicability of NiCo(OH)_2_.rGO/GCE was tested in food samples such as biscuit and chocolate samples using SWV (Figure 7A,B). The chocolate and biscuit samples were grinded to a fine powder in an agate mortar. Then, 1.0 g of the powder sample was dispersed in 5% ethanol and 95% buffer solution (pH = 7.2), followed by sonication treatment for 1 h and then filtration. The sample extracts were further diluted in 0.1 M PBS (pH = 7.2). The standard addition method was followed in this study to minimize the matrix effect that interferes with the measurement of the analyte. A known concentration of VAN was spiked into the food samples, and the electrocatalytic performance was evaluated. The oxidative current was utilized to calculate the recovery percentage and RSD. The recovery results are summarised in Table 2, which shows that the recovery was in the range of 99.8–100.7%, with the relative standard deviations of 3.12% and 3.17% for the biscuit and chocolate samples, respectively. These results indicate that our designed sensor is promising for real sample analysis.

## 4. Conclusions

In summary, we have demonstrated a straightforward and effective method for the fabrication of a Ni and Co bimetallic hydroxide and rGO nanocomposite (NiCo(OH)_2_.rGO/GCE). Our synthesized material acted as an efficient sensor for the electrochemical determination of VAN. It exhibited high stability, selectivity, and sensitivity (631.2 nA nM^−1^ cm^−2^), as well as a low limit of detection (6.1 nM). Most notably, the LOD obtained using our modified sensor was comparable with those reported in the literature. Lastly, the practical applicability of our designed material was evaluated for the determination of VAN in biscuit and chocolate samples. In conclusion, the designed sensor is a suitable candidate for industrial applications, especially for the detection of vanillin in food samples.

## Figures and Tables

**Table 1:** Comparison table for the electrochemical determination of VAN using NiCo(OH)_2_.rGO/GCE with some previously reported sensors.

Electrode	Media	Method	Linear Rang (µM)	LOD (µM)	Ref.
Au-FrGO/GCE	0.1 M PBS	DPV	1–150	0.15	[23]
Graphene Nanoflake/GCE	0.1 M PBS	DPV	0.01–53	0.012	[44]
CoS nanorods/GCE	0.025 M H_2_SO_4_	DPV	0.5–56	0.07	[45]
AuNP-PAH/GCE	0.2 M ABS	SWV	0.9–15	0.05	[46]
Lysine/CPE	0.1 M PBS	DPV	10–100	2.88	[47]
AuPd–graphene/GCE	0.1 M PBS	DPV	10–40	0.02	[48]
Gr/GCE	0.1 M PBS	DPV	0.6–48	0.05	[49]
MoS_2_-CNF/GCE	0.1 M PBS	CA	0.3–135	0.15	[50]
TBAC-900/GCE	0.1 M PBS	DPV	5–1150	0.68	[51]
CNT-SPE	0.05 M BRS	DPV	2.5–750	1.03	[52]
**NiCo(OH)_2_.rGO** **/GCE**	**0.1 M PBS**	**SWV**	**0.005–0.14**	**0.006 µM**	**This Work**

**Table 2:** Real sample detection of VAN in biscuit and chocolate samples.

Sample	Added (nM)	Found (nM)	Recovery (%)	RSD (%)
Biscuit	0	ND	-	-
	5.0	5.01	100.2	
	10.0	10.03	100.3	3.12
	15.0	14.97	99.8	
	20.0	20.15	100.7	
Chocolate	0	ND	-	-
	5.0	5.03	100.6	
	10.0	9.99	99.9	3.17
	15.0	15.02	100.1	
	20.0	20.09	100.4	

ND = Not detected.

## Data Availability

Data are contained within the article.
